# The Impact of Resident Involvement on Postoperative Complications After Shoulder Arthroscopy: A Propensity-Matched Analysis

**DOI:** 10.5435/JAAOSGlobal-D-20-00138

**Published:** 2020-09-14

**Authors:** Trevor R. Gulbrandsen, Zain M. Khazi, Alan G. Shamrock, Qiang An, Kyle Duchman, J. Lawrence Marsh, Robert W. Westermann, Brian Wolf

**Affiliations:** From the Department of Orthopedics and Rehabilitation, University of Iowa Hospitals and Clinics, Iowa City, IA.

## Abstract

Shoulder arthroscopy is the second most frequently performed procedure by orthopaedic surgeons taking the American Board of Orthopaedic Surgery part II examination. However, the impact of resident involvement on outcomes after shoulder arthroscopy is poorly understood. The aim of this study was to investigate whether resident involvement in shoulder arthroscopic procedures affects postoperative complication rates and surgical time using propensity score-matched cohorts.

**Methods:**

The American College of Surgeons National Surgical Quality Improvement Program registry was queried to identify patients who underwent common shoulder arthroscopic procedures between 2006 and 2012. Cases without information on resident involvement, treatment of septic arthritis or osteomyelitis of the shoulder, or concomitant open or miniopen procedures were excluded from the study. A 1:1 propensity score match was used based on demographic and comorbidity factors to match cases with resident involvement to nonresident cases. Patient demographics, comorbidities, surgical time, length of hospital stay, and 30-day postoperative complications were compared between the two groups.

**Results:**

Overall, 15,857 patients who underwent shoulder arthroscopy were identified. After propensity score matching, 3474 cases (50% with resident involvement) were included. Appropriate matching was verified with no difference in demographic or health characteristics. No significant differences in the overall rate of 30-day complications was noted in resident-involved versus nonresident group (*P* = 0.576). No significant difference was observed in postoperative surgical or medical complications. Resident involvement was significantly longer surgical time (75.9 ± 35.9 versus 75.1 ± 40.5 minutes, *P* = 0.03) when compared with cases performed without a resident.

**Conclusions:**

Resident involvement in shoulder arthroscopy is not associated with increased risk for medical or surgical 30-day postoperative complications. Resident participation in shoulder arthroscopy cases did increase surgical time; however, this finding is likely clinically insignificant.

Resident participation in surgical procedures is vital during orthopaedic training. Arthroscopy is especially important because previous studies have found that shoulder arthroscopy is the second most common procedure performed by orthopaedic surgeons taking the American Board Orthopaedic Surgery (ABOS) part II examination.^1^ The Residency Review Committee in Orthopaedic Surgery considers shoulder arthroscopy a core competency and requires residents to meet the benchmark shoulder arthroscopic case minimums before graduation.^2^ It is important to know whether the risk of postoperative complications is increased when residents participate in shoulder arthroscopy procedures.

Surgeon educators must assess both resident skill and surgical case complexity when determining how residents will participate in a surgical case. The duration of the procedure, patient safety, and cost effectiveness are all important considerations. General surgery, ophthalmology, neurosurgery, and urology have evaluated complications when residents are involved,^3,4,5,6,7,8^ with various procedures, but the impact on complications when residents participate in shoulder arthroscopy procedures has not been studied well.

The purpose of the current study was to investigate whether resident involvement in shoulder arthroscopic procedures affects short-term postoperative complication rates and surgical time in propensity score-matched cohorts. We hypothesize that resident involvement is not associated with increased postoperative complication rates or surgical time after shoulder arthroscopy.

## Methods

### Data Source

The American College of Surgeons National Surgical Quality Improvement Program database (NSQIP) was analyzed from 2006 to 2012 to identify patients undergoing shoulder arthroscopy procedures. The NSQIP prospectively collects over 300 preoperative and postoperative patient variables from over 600 private and academic hospitals across the United States.^9^ This information is collected by trained clinical reviewers for up to 30 days after surgery. Information collected in the NSQIP includes patient demographics, medical comorbidities, intraoperative information, additional surgeries, and postoperative complications within 30 days of surgery. Patient records were retrieved and analyzed using the Current Procedural Terminology (CPT) and International Classification of Diseases Ninth Revision codes. Cases are selected based on a Health Insurance Portability and Accountability Act compliant systematic sampling process with a reported interobserver disagreement rate of 2%.^9,10^ Owing to the data being acquired in a Health Insurance Portability and Accountability Act compliant manner, this study was granted exemption from the local institutional review board.

### Patient Selection and Determining Resident Involvement

**Figure 1:**
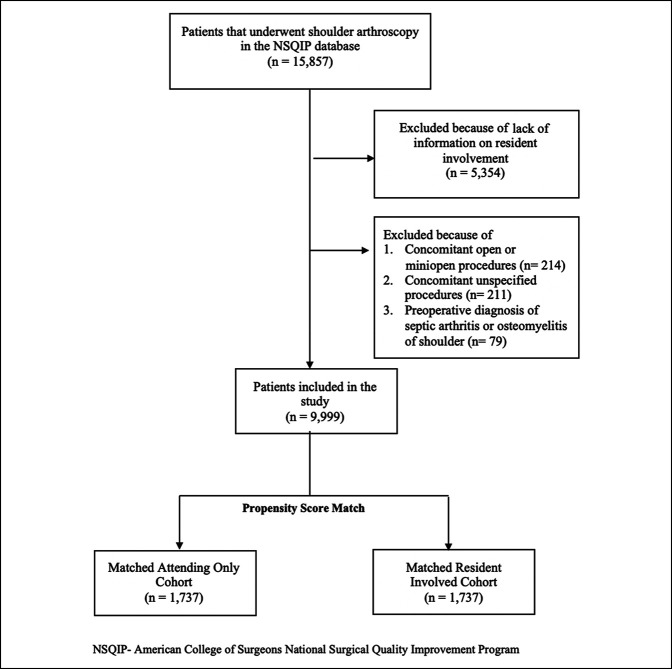
Flowchart of inclusion and exclusion criteria. NSQIP = National Surgical Quality Improvement Program

From 2006 to 2012, 15,857 shoulder arthroscopy cases were identified using relevant CPT codes (Table 1). The study period was limited to 2006 to 2012 because the NSQIP only collected information regarding resident involvement before 2013. Of the cases identified in the database, 5354 cases (33.8%) were initially excluded because of lack of information regarding resident involvement in the case. Furthermore, 425 cases (2.7%) were excluded because of concomitant open or miniopen procedures such as open rotator cuff repair, open biceps tenodesis, or mass excisions. In addition, 79 cases (0.5%) were excluded because of preoperative diagnosis of septic arthritis of the shoulder or osteomyelitis around the shoulder joint. As a result, before matching, only 9999 cases were further analyzed, of which 1798 (18%) were performed with a resident in the operating room (Figure 1).

**Table 1:** Shoulder Arthroscopy CPT Codes

CPT code	Description	n, (%)
29806	Shoulder arthroscopy, capsulorrhaphy	755 (7.6)
29807	Shoulder arthroscopy, repair of SLAP lesion	988 (9.9)
29819	Shoulder arthroscopy, removal of loose or foreign body	48 (0.5)
29820	Shoulder arthroscopy, partial synovectomy	28 (0.3)
29821	Shoulder arthroscopy, complete synovectomy	55 (0.6)
29822	Shoulder arthroscopy, limited débridement	377 (3.8)
29823	Shoulder arthroscopy, extensive débridement	442 (4.4)
29824	Shoulder arthroscopy, distal claviculectomy (the Mumford procedure)	544 (5.4)
29825	Shoulder arthroscopy, lysis of adhesions with or without manipulation under anesthesia	159 (1.6)
29826	Shoulder arthroscopy, decompression of subacromial space with partial acromioplasty	2967 (29.7)
29827	Shoulder arthroscopy, rotator cuff repair	3532 (35.3)
29828	Shoulder arthroscopy, biceps tenodesis	118 (1.2)

CPT = Current Procedural Terminology code, SLAP = superior labral tear from anterior to posterior

### Preoperative Demographics and Comorbidities

Basic patient demographics including age, sex, and race (Caucasian, Black, or other) are recorded in the database and were compared between resident-involved and no resident involvement cohorts. Preoperative comorbidities such as obesity were calculated from each patients' height and weight. Other preoperative conditions including diabetes mellitus, smoking history, chronic obstructive pulmonary disease, and the American Society of Anesthesiologist (ASA) classification were compared between the two cohorts. Diabetes mellitus is categorized as insulin-dependent, noninsulin-dependent, or no diabetes in the database; however, for the purposes of this study, the patients were categorized as having diabetes mellitus (insulin-dependent or noninsulin-dependent) or not. Similarly, smoking status was categorized as current smoker, former smoker, or nonsmoker; however, in this study, patients were categorized as having a history of smoking tobacco (current or former smoker) or nonsmoker. In addition to preoperative comorbidities, certain preoperative laboratory values are also recoded in the NSQIP. For the purposes of this study, laboratory values for preoperative albumin, creatinine, hematocrit, international normalized ratio, platelet count, and white blood cell count were compared between resident-involved and nonresident cohorts (Table 2).

**Table 2:** Comparison of Patient Demographics and Comorbidities Between Resident Involved and Nonresident Cohorts

Patient Characteristics	Nonresident			Resident Involved			*P* Value	
	Unadjusted (n = 8705)		Matched (n = 1737)	Unadjusted (n = 1798)		Matched (n = 1737)	Unadjusted, *P* Value	Matched, *P* Value
Demographics and comorbidities								
Age, mean (SD)	51.72 (15.02)		53.04 (14.9)	52.58 (14.85)		52.74 (14.76)	**0.0219**	0.5776
Female sex	4491 (40.1)		708 (40.8)	731 (40.8)		711 (40.9)	0.5959	0.9175
Race/ethnicity								
Caucasian	6517 (74.9)		1312 (75.5)	1175 (65.4)		1137 (65.5)	**<0.0001**	**<0.0001**
Black	579 (6.7)		129 (7.4)	135 (7.5)		127 (7.3)		
Other	1609 (18.5)		296 (17.0)	488 (27.1)		473 (27.2)		
Obesity, BMI >30 kg/m^2^, mean (SD)	29.7 (6.6)		29.4 (6.3)	29.6 (7.0)		29.5 (6.3)	0.0729	0.9070
Diabetes mellitus	1050 (12.1)		189 (10.9)	205 (11.4)		201 (11.6)	0.4318	0.5190
Smoking	1679 (19.3)		288 (16.6)	307 (17.1)		300 (17.3)	**0.0316**	0.5872
Chronic obstructive pulmonary disease	198 (2.3)		48 (2.8)	39 (2.2)		39 (2.3)	0.7840	1.3285
The American Society of Anesthesiologist classification								
1-No disturbance	1524 (17.6)	282 (16.2)		306 (17.0)		287 (16.5)	0.0634	0.3745
2-Mild disturbance	5147 (59.3)	1057 (60.9)		1070 (59.5)		1046 (60.2)		
3-Severe disturbance	1972 (22.7)	389 (22.4)		403 (22.4)		386 (22.2)		
4-Life-threatening	42 (0.5)	9 (0.5)		18 (1.0)		18 (1.0)		
Mean preoperative laboratory values								
Hematocrit, mean (SD)	41.5 (4.11)	41.6 (4.05)		41.13 (4.13)	41.17 (4.12)		**0.0143**	0.0645
White blood cell count, mean (SD)	7.08 (2.3)	7.08 (2.83)		7.17 (2.46)	7.17 (2.47)		0.8947	0.4233
Platelet count, mean (SD)	245.41 (66.67)	242.54 (64.3)		239.19 (67.91)	238.6 (67.38)		**0.0039**	0.1619
INR, mean (SD)	1.05 (0.34)	1.04 (0.22)		1.12 (0.47)	1.09 (0.34)		0.3374	0.3139
Albumin, mean (SD)	4.19 (0.43)	4.20 (0.43)		4.1 (0.40)	4.1 (0.39)		**0.0001**	**0.0024**
Creatinine, mean (SD)	0.94 (0.45)	0.95 (0.26)		0.93 (0.43)	0.93 (0.42)		0.2489	**0.0024**

BMI = body mass index, INR = international normalized ratio

Bold: significant finding (*P* < 0.05).

### Outcomes

The NSQIP database tracks patients for any occurrence of various adverse events for 30 days postoperatively. Therefore, only 30-day postoperative complications were compared between the cohorts. The primary outcomes evaluated were surgical complications, medical complications, surgical time, and length of hospital stay. In this study, surgical complications were defined as any occurrence of superficial surgical site infection (SSI), deep SSI, wound dehiscence, intraoperative or postoperative blood transfusions, and neurologic deficits. Medical complications were defined as any occurrence of pulmonary embolism, deep vein thrombosis, urinary tract infection, renal insufficiency, myocardial infarction, >48 hours of ventilation, cerebrovascular incident, pneumonia, or sepsis.

### Statistical Analysis

Retrospective analysis may be confounded by selection bias; therefore, a propensity score-matching algorithm was used to create similar groups for the resident-involved and nonresident-involved cohorts. In this study, a 1:1 propensity score match was used based on age, sex, body mass index, obesity, smoking history, and ASA classification to match cases with resident involved to no resident involvement cases. The matched cohorts were compared for preoperative patient demographics, comorbidities, laboratory values, and postoperative complications using Fisher exact test or Pearson Chi-square test for categorical variables and Students *t*-tests for continuous variables. In addition, a multivariate Poisson analysis was also performed to compare surgical time and length of hospital stay between the cohorts. Finally, a post hoc analysis was also performed to assess the power and the effect size of the study. All statistical analyses were performed using SAS version 9.4 (Cary, NC) with statistical significance set at *P* < 0.05.

## Results

### Patient Characteristics

After propensity score matching, 3474 cases (50% with resident involvement) were included in the study. Both matched cohorts were similar in age (*P* = 0.578), sex (*P* = 0.918), body mass index (BMI) (*P* = 0.907), diabetes mellitus (*P* = 0.519), chronic obstructive pulmonary disease (*P* = 0.329), smoking history (*P* = 0.588), and ASA classification (*P* = 0.375), which confirmed an appropriate match (Table 2). Both groups were similar in preoperative laboratory test results except for albumin levels (nonresident cohort: 4.20 ± 0.43; resident Involved: 4.10 ± 0.39, *P* = 0.002) and creatinine (nonresident cohort: 0.95 ± 0.26; resident involved: 0.93 ± 0.42, *P* = 0.006).

### Postoperative Complication Rates

The overall incidence of 30-day complications was similar in the nonresident- (0.92%) and resident-involved (0.75%) groups (*P* = 0.576; Table 3). No significant difference was noted in postoperative surgical complications including SSI (*P* = 1.000), deep SSI (*P* = 1.000), neurologic deficit (*P* = 1.000), or blood transfusion (*P* = 1.000). Furthermore, no significant difference was observed in postoperative medical complications including pulmonary embolism (*P* = 0.2183), deep vein thrombosis (*P* = 0.2498), urinary tract infection (*P* = 1.000), pneumonia (*P* = 1.000), or sepsis (*P* = 1.000).

**Table 3:** Comparison of 30-day Complication Rates Between Nonresident- and Resident-Involved Groups

Complications	Nonresident Cohort, n = 1737(%)	Resident, n = 1737 (%)	*P* Value
Surgical time (min)	75.1 ± 40.5	75.9 ± 35.9	**0.034**
Overall complication rate	16 (0.92)	13 (0.75)	0.576
Surgical complications			
Superficial surgical site infection	3 (0.17)	2 (0.12)	1.000
Deep surgical site infection	0 (0)	1 (0.06)	1.000
Neurologic deficit	0 (0)	1 (0.06)	1.000
Blood transfusion	1 (0.06)	2 (0.12)	1.000
Medical complications			
Pneumonia	1 (0.06)	2 (0.12)	1.000
Urinary tract infection	3 (0.17)	2 (0.12)	1.000
Sepsis	0 (0)	1 (0.06)	1.000
Deep vein thrombosis	3 (0.17)	0 (0)	0.2498
Pulmonary embolism	5 (0.29)	2 (0.12)	0.2183

Bold: significant finding (*P* < 0.05).

### Poisson Regression Model

Analysis of surgical time and length of hospital stay was performed using a Poisson regression model. Shoulder arthroscopy cases with resident involvement had significantly longer surgical times (resident involved: 75.9 ± 35.9 minutes; surgeon only: 75.1 ± 40.5 minutes, *P* = 0.034) when compared with cases performed without a resident (Table 4). No difference was observed in the length of hospital stay for cases with a resident versus cases performed without a resident (resident involved: 0.23 ± 2.42 days; surgeon only: 0.24 ± 2.79 days, *P* = 0.295).

**Table 4:** Poisson Regression Analysis for Surgical Time and Length of Hospital Stay

Variable	Nonresident Cohort	Resident Involved	*P* Value
	n = 1447	n = 1447	
Surgical time, min (SD)	75.1 ± 40.5	75.9 ± 35.9	**0.0340**
Length of hospital stay, days (SD)	0.24 ± 2.79	0.23 ± 2.42	0.2951

Bold: significant finding (*P* < 0.05).

## Discussion

The number of shoulder arthroscopic procedures being performed in the United States is increasing.^11^ Therefore, it is essential for orthopaedic residents to have adequate involvement and training with these procedure. This study compared matched patient cohorts, from the NSQIP database, who underwent shoulder arthroscopy with a resident involved compared with the procedure solely being performed by practicing surgeon without resident involvement. We identified an overall low 30-day complication rate (<0.92%) with no notable difference in surgical or medical complications between the two groups. A statistically significant, however, clinically insignificant difference was observed in surgical time (*P* = 0.034), with longer procedures performed in the resident-involved cohort. Overall, this study demonstrates that resident involvement does not increase perioperative morbidity after shoulder arthroscopy.

The low complication rate demonstrated in this study is similar to previous studies investigating the overall complication rate of shoulder arthroscopy. Using the NSQIP database, Shields et al.^12^ similarly identified a short-term complication rate of 1% with a 0.57% rate of major complications and 0.53% rate of minor complications after shoulder arthroscopy. Similarly, Rubinstein et al.^13^ reported a 1.6% incidence of adverse events after shoulder arthroscopy in patient aged 60 years and older. Shin et al.^14^ examined complications associated with shoulder arthroscopy in recently trained surgeons by using the ABOS database. They performed a thorough study by evaluating 27,072 shoulder arthroscopy procedures performed by surgeons who underwent the ABOS part II examination from 2012 to 2016. They reported the rates of surgical, medical, and anesthetic complications to be 7.9%, 2.2%, and 1.0%, respectively in arthroscopic shoulder procedures. Although the surgical and medical complications were higher than other studies, including the present one, they specifically evaluated less experienced surgeons and used a larger database that included complications that the NSQIP does not, including postoperative stiffness/arthrofibrosis. In addition, the ABOS database complications are self-reported by the surgeons. Overall, the literature demonstrates a low incidence of complications after shoulder arthroscopy, and the findings of this study provide further support.

Proper resident education and training is essential for the future of healthcare. With the combination of work hour restrictions and increased diversity of orthopaedic procedures and subspecialization, residency programs must seek optimal procedural exposure for their residents to prepare them for independent practice. It is crucial for residents to be involved in core orthopaedic surgical cases to develop these skills. The impact of resident involvement in surgical procedures has been explored in various surgical and orthopaedic procedures.^15,16,17,18,19,20,21,22^ Haughom et al.^21^ performed a retrospective analysis on the impact of residents involved in total hip arthroplasty^20^ and total knee arthroplasty cases. These two studies did not identify resident involvement as a risk factor for 30-day morbidity or mortality in these selected cases. Cvetonovich et al. performed a propensity score analysis for patients undergoing total shoulder arthroplasty and reported no evidence of increased 30-day complications or mortality with associated resident involvement.^19^ More recently, Lebedeva et al.^15^ confirmed these findings of low complication rates with no impact of resident involvement in patients undergoing anterior cruciate ligament reconstruction.

Using the NSQIP database, Basques et al.^23^ investigated resident involvement in shoulder arthroscopy. They identified a 1.09% overall complication rate with resident involvement not being associated with complications, adverse events, or 30-day readmission. No reported data were observed on the differences of the length of hospital stay. They did not determine a notable difference in surgica; times between cases involving residents and those without a resident. This study also identified a low overall complication rate of 0.92% and did not find notable differences in postoperative complications, length of hospital stay, or adverse events between shoulder arthroscopies with and without residents involved. By contrast, this study determined a statistically significant difference in surgical times with resident involved cases being marginally longer on average. However, this finding is likely clinically insignificant because the resident involved cohort took 45 seconds longer on average. This minimal difference can be due to various reasons including referral of complex cases to academic centers, which can be difficult to control for using the database.

The findings of this study differ slightly from the findings by Basques et al. because of differences in statistical analysis methods. Basques et al. performed a multivariate Poisson regression analysis for all 15,774 patients to compare the rates of postoperative complications between the two cohorts. By contrast, this study had a more narrow inclusion criteria and used a propensity score analysis to match patients in resident involved and nonresident cohorts.

Propensity score matching was introduced in 1983 and is a complementary method when randomization is not possible.^24^ The propensity score is the probability of the assigned treatment that is conditional on certain baseline characteristics. Through this technique, a balancing score is created, and therefore, the baseline covariates between the two comparable cohorts will be similar. This allows for one to perform a nonrandom observational study that is designed to contain certain analytic characteristics of a randomized controlled trial. Although propensity score balancing methods do not allow causal conclusions, the elimination of various confounding variables can be performed, whereas regression-based approaches do not provide this same assurance.^25,26^ With concerns of the risk of selection bias, because Basques et al. cohort consisted only 12.3% cases involving a resident, we elected to perform propensity score analysis. Through this matching algorithm, the cohort was narrowed down to 3474 cases (50% with resident involvement). An analysis confirmed appropriate matching because both groups were similar in age (*P* = 0.578), sex (*P* = 0.918), BMI (*P* = 0.907), diabetes mellitus (*P* = 0.519), chronic obstructive pulmonary disease (*P* = 0.329), smoking history (*P* = 0.588), and ASA classification (*P* = 0.375). Further analysis in this matching cohort demonstrated safe and timely patient outcomes when residents are involved in shoulder arthroscopy procedures.

There were several limitations with this study. First, the retrospective analysis limits causal conclusions and only associations can be ascertained. In addition, several confounders are observed that cannot be accounted for, even with propensity score matching. Academic teaching hospitals are often tertiary centers and treat patients with complex orthopaedic problems, which alters both the operative and postoperative care of the patients. The NSQIP does not report orthopaedic-relevant issues including case-specific factors, previous surgery, or the degree of injury and only uses general CPT codes. Therefore, we cannot perform propensity match analysis based on case complexity. Furthermore, the NSQIP data have its own limitations, including the inability to assess and control for specific radiographic findings or soft-tissue injuries. In addition, NSQIP does not detail the extent of resident involvement during the surgery and only if the resident was listed during the case. Only 30-day complication rates are reported; therefore, this study is unable to assess medium- and long-term complications, patient-reported outcomes, or functional status of the patient postoperatively. This includes orthopaedic-specific outcomes. Finally, the NSQIP is the largest available database with information regarding resident involvement during surgical procedures. Therefore, the lack of short-term adverse impact of resident involvement after shoulder arthroscopy may be secondary to a type II error. According to our post hoc analysis, the power of this study was low at 0.09. However, the calculated effect size for resident involvement and adverse events was very low, suggesting that if a relationship exists, then this finding is unlikely to be clinically significant. Despite these limitations, the NSQIP database and the statistical analysis performed provide a sound analysis on the impact of resident involvement on postoperative complications and surgical times for shoulder arthroscopy.

## Conclusions

Resident involvement in shoulder arthroscopy procedures is not associated with increased risk for medical or surgical 30-day postoperative complications. Overall, this study demonstrated no increased perioperative morbidity associated with resident surgical involvement.
